# Theoretical insights into selective electrochemical conversion of carbon dioxide

**DOI:** 10.1186/s40580-019-0177-2

**Published:** 2019-03-12

**Authors:** Chan Woo Lee, Chanyeon Kim, Byoung Koun Min

**Affiliations:** 1 Clean Energy Research Center, Korea Institute Science and Technology, Seoul, 02792 Republic of Korea; 2 Department of Applied Chemistry, Kookmin University, Seoul, 02707 Republic of Korea; 3 Green School, Korea University, Seoul, 02841 Republic of Korea

**Keywords:** Electrocatalysis, CO_2_ reduction, Intermediate binding energy, Theoretical calculation

## Abstract

Electrochemical conversion of CO_2_ and water to valuable chemicals and fuels is one of the promising alternatives to replace fossil fuel-based processes in realizing a carbon–neutral cycle. For practical application of such technologies, suppressing hydrogen evolution reaction and facilitating the activation of stable CO_2_ molecules still remain major challenges. Furthermore, high production selectivity toward high-value chemicals such as ethylene, ethanol, and even *n*-propanol is also not easy task to achieve. To settle these challenges, deeper understanding on underlying basis of reactions such as how intermediate binding affinities can be engineered at catalyst surfaces need to be discussed. In this review, we briefly outline recent strategies to modulate the binding energies of key intermediates for CO_2_ reduction reactions, based on theoretical insights from density functional theory calculation studies. In addition, important design principles of catalysts and electrolytes are also provided, which would contribute to the development of highly active catalysts for CO_2_ electroreduction.

## Introduction

Climate change due to greenhouse gases has been a critical issue that must be solved for human being’s sustainable life [1]. Indiscriminate exploitation of fossil fuel has accelerated the global warming issue associated with greenhouse gas emission [2], therefore, clean energy generation technologies should be developed to mostly or partly replace the fossil fuel-based processes. Electrochemically converting CO_2_ and water to valuable chemicals and fuels, using renewable energy resources such as solar energy, has been one of the promising strategies to realize carbon–neutral energy cycle [3, 4]. It can generate a variety of products including HCOOH, CO, CH_4_, C_2_H_4_, C_2_H_5_OH, and even C_3_–C_4_ chemicals under ambient conditions in a sustainable fashion [5–9]. However, for practical implementation of the electrochemical CO_2_ conversion technology, suppressing hydrogen evolution reactions (HER) and enhancing selectivity for specific products remain challenges. In general, the HER, a proton reduction reaction, easily occurs as a competing reaction of CO_2_ reduction reactions (CO_2_RR) because it is kinetically more facile [10]. In addition, the CO_2_ can be reduced to various intermediates and products via multiple reaction pathways, resulting in poor selectivity of the reaction [6].

In the electrochemical reaction system, product selectivity of CO_2_RR is deeply associated with the binding affinity of reaction intermediates on the catalyst surface. In specific, polycrystalline Au, Ag and Zn electrodes can evolve CO with high selectivity because these metals intrinsically possess low binding affinities for an adsorbed *CO intermediate [10–15]. In contrast, Pt, Ni, and Fe electrodes are poisoned by *CO owing to their strong binding affinities for *CO [16]. As a result, HER proceeds with high selectivity over CO_2_RR. Meanwhile, high selectivity for HCOO^−^ production have been shown from the metals like Sn, Pb and Bi that relatively favor *OCHO over *COOH [17–19]. Therefore, engineering or controlling binding affinities of key intermediates is a critical issue in the product selectivity enhancement.

Recently, effective strategies to tune intermediate binding affinities have been suggested and demonstrated by several pioneering works. Representatively, it was reported that electrochemically reduced Cu oxides possess subsurface oxygens near the catalyst surface, which can increase the *CO binding affinity because the oxygens withdraw electrons from the Cu sp-band and reduce the σ-repulsion between a Cu atom and *CO molecule [20]. As a consequence, C–C coupling reactions by CO dimerization at the catalyst surface with high CO coverage can be kinetically promoted. Such a stronger CO binding through subsurface oxygens was also theoretically rationalized in Ag case [21]. In addition, it has been suggested that nanoneedle morphology can enhance electric field at the tip of nanostructure, thereby the alkali metal ions can be concentrated [22]. The solvated alkali metal cations selectively stabilize the polarizable intermediates like *CO_2_, *CO, *OCCO and *OCCHO due to the electrostatic interactions while *H is insensitive to the cations due to negligible polarizability [23]. As a result, higher CO_2_RR selectivity could be achieved.

Here, we first address intrinsic limitations of conventional pure metals in terms of binding affinity and product selectivity. Then, we review recent strategies to allow for the binding affinity modulation of CO_2_ reduction intermediates. The strategies are classified into five groups: (1) oxygen incorporation, (2) electrolyte engineering, (3) nanostructuring, (4) introducing surface ligands, and (5) single-atom catalyst. In each subject, we cover theoretical insights into how the modifications can induce the tuning of binding affinity based on density functional theory (DFT) calculation studies. This review provides important design principles of electrocatalysts to promote CO_2_RR over HER, as well as C–C coupling via CO dimerization pathway.

## Challenges of electrochemical CO_2_ reduction reactions

CO_2_ is a stable molecule (ΔG_f_ = − 394.4 kJ/mol) that has electrophilic carbon center due to neighboring oxygen atoms with a linear configuration. The one-electron transfer to CO_2_ to form the CO_2_^−^ anion, an initially created intermediate in most cases, requires a very negative potential of − 1.9 V vs. SHE in a neutral aqueous solution (Table 1) [24]. This thermodynamic limitation makes the overpotential for CO_2_RR much larger than HER, leading to low selectivity for CO_2_RR. Furthermore, although the CO_2_ molecule can be activated at more favorable overpotentials by the concerted proton-electron transfer pathway or the promotional effect of alkali metal cations contained in electrolytes [25, 26], a criticial issue is laid on controlling product selectivity. The multiple CO_2_ reduction reactions that produce different chemicals including HCOO^−^, CO, CH_4_, C_2_H_4_ and C_2_H_5_OH can occur concurrently as described in Table 1. In addition, HER acts as a major competing reaction. The HER is kinetically more facile than CO_2_RR because the number of electrons involved in the reaction is only two, and because the concentrations of proton sources such as water and bicarbonate ions are much higher than that of CO_2_ in the neutral or basic aqueous solutions frequently used in CO_2_RR experiments [27]. Due to the thermodynamic as well as kinetic reasons, selectively converting CO_2_ to desirable products at low overpotential is challenging. To overcome the issues, we need to deeply understand crucial factors that affect the CO_2_RR properties.

**Table 1:** Standard reduction potential of electrochemical CO_2_ reduction

Half reaction	Number of electron	Potential (V vs. SHE) at pH 7
CO_2_ (g) + e^−^ → *COO^−^	1	− 1.90
CO_2_ (g) + 2H^+^ + 2e^− ^→ HCOOH (l)	2	− 0.61
CO_2_ (g) + H_2_O (l) + 2e^−^ → HCOO^−^ (aq) + OH^−^	2	− 0.43
CO_2_ (g) + 2H^+^ + 2e^−^ → CO (g) + H_2_O (l)	2	− 0.53
CO_2_ (g) + H_2_O (l) + 2e^−^ → CO (g) + 2OH^−^	2	− 0.52
CO_2_ (g) + 4H^+^ + 4e^−^ → HCHO (l) + H_2_O (l)	4	− 0.48
CO_2_ (g) + 3H_2_O (l) + 4e^−^ → HCHO (g) + 4OH^−^	4	− 0.89
CO_2_ (g) + 6H^+^ + 6e^−^ → CH_3_OH (l) + H_2_O (l)	6	− 0.38
CO_2_ (g) + 5H_2_O (l) + 6e^−^ → CH_3_OH (l) + 6OH^−^	6	− 0.81
CO_2_ (g) + 8H^+^ + 8e^−^ → CH_4_ (g) + 2H_2_O (l)	8	− 0.24
CO_2_ (g) + 6H_2_O (l) + 8e^−^ → CH_3_OH (l) + 8OH^−^	8	− 0.25
2CO_2_ (g) + 12H^+^ + 12e^−^ → C_2_H_4_ (g) + 4H_2_O (l)	12	0.06
2CO_2_ (g) + 8H_2_O (l) + 12e^−^ → C_2_H_4_ (g) + 12OH^−^	12	− 0.34
2CO_2_ (g) + 12H^+^ + 12e^−^ → C_2_H_5_OH (g) + 3H_2_O (l)	12	0.08
2CO_2_ (g) + 9H_2_O (l) + 12e^−^ → C_2_H_5_OH (g) + 12OH^−^	12	− 0.33

Previously, Hori et al. [16] have shown that the product selectivity changes with the type of transition metal electrodes, from the systematic investigation on the CO_2_RR activity of various metal foils. They categorized transition metals into four groups on the basis of product distribution. The first group consists of metals producing H_2_ gas dominantly like Pt, Ir, Fe, Co and Ni metals. The second group includes the metals of which primary product is CO such as Au, Ag and Zn. Third group includes metals such as Cd, In, Sn, etc. and their primary product is HCOO^−^. The last group contains only Cu which is a unique single metal that can produce various hydrocarbons and alcohols including CH_4_, C_2_H_4_, C_2_H_5_OH and C_3_H_7_OH.

**Fig. 1:**
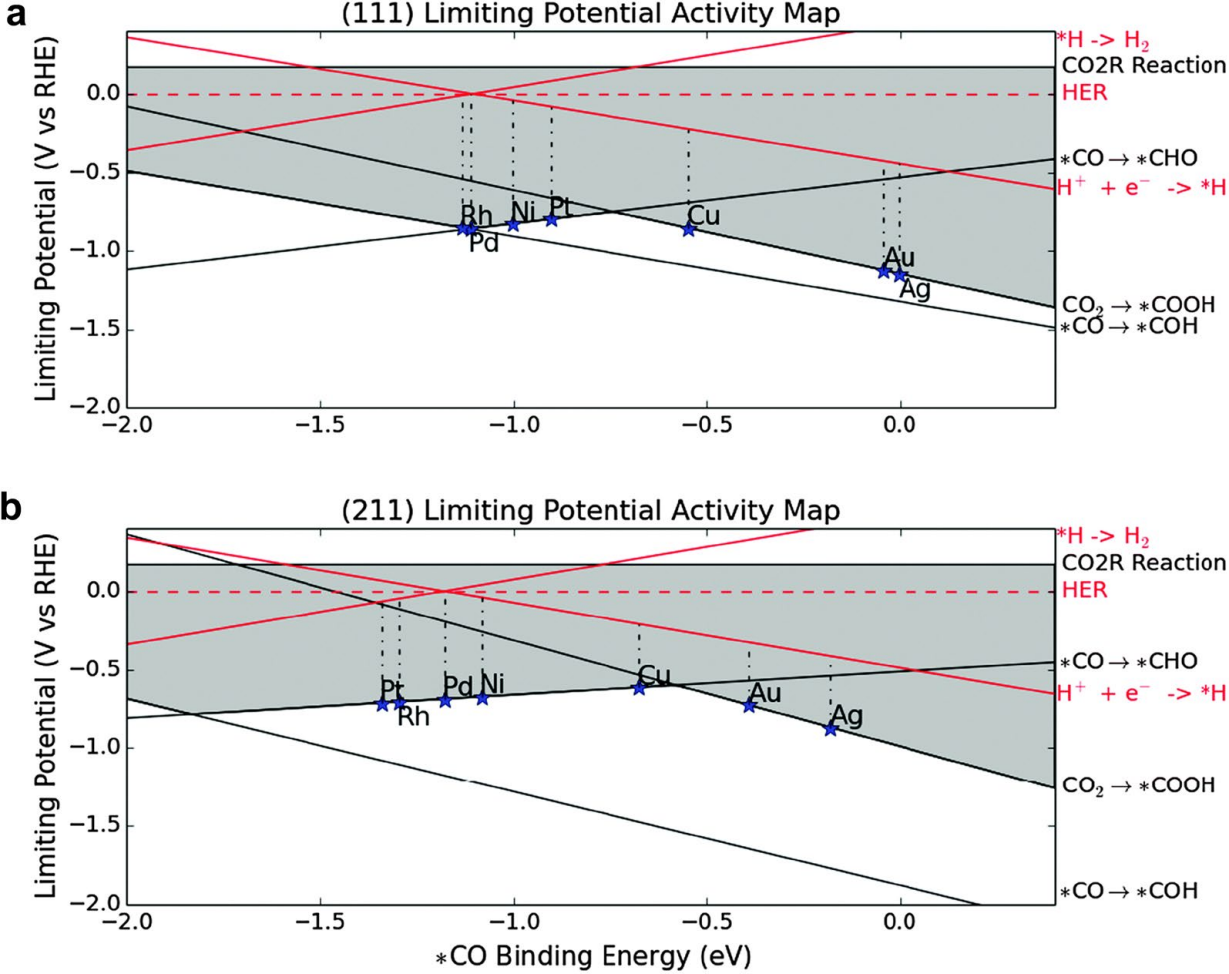
Limiting potential activity map of CO_2_ reduction on the **a** (111) and **b** (211) surface of FCC transition metals (Reproduced with permission from [30], copyright 2014 Royal Society of Chemistry)

Recently, the experimental trend firstly demonstrated by Hori et al. could be understood from DFT calculation studies. The theoretical simulation results revealed that the intrinsic CO_2_RR selectivity on various metal surfaces is closely related with the binding energy of reaction intermediates [28–30]. Norskov group explained that Au and Ag metals, representative catalysts for CO production, have weak *CO binding energy, which allows for *CO desorption rather than further hydrogenation toward *CHO and *COH as shown in Fig. 1 [30]. Additionally, the Au and Ag metal can show a low catalytic activity for HER because of the weak binding energy of *H. In contrast, the Ni, Pt, Pd and Rh metals which dominantly produce H_2_, have too strong *CO binding energy to evolve *CO, whereas HER is facilitated due to their ideal *H binding energy based on Sabatier principle. Exceptionally, the Cu metal has a medium *CO binding energy and relatively unfavorable limiting potential for HER. Thereby, further hydrogenation of *CO intermediates can proceed at the catalyst surface, generating various hydrocarbons and alcohols.

While the binding energy of the key reaction intermediates can give us important insights as a descriptor to understand intrinsic activity of various metals, enhancing their catalytic properties such as selectivity and overpotential is limited by scaling relations between the binding energies of various intermediates. For instance, the binding energies for *COOH and *CO intermediates are correlated on transition metals by the equation, ΔG_CO_ = 1.31 × ∆G_COOH_ − 0.47 [5]. Thus, the binding energies are hard to be controlled independently although stronger binding of *COOH and weaker binding of *CO is required to efficiently promote CO production. In addition, the scaling relation also exists between the intermediate binding energies of *COOH and *H based on the equation, ΔG_COOH_ = 1.65 × ∆G_H_ + 0.02 [5]. which also makes it difficult to selectively suppress HER. In this regard, breaking these scaling relations is critical in enhancing the catalyst properties for CO_2_RR. In the following chapter, we outline recent strategies that allow for the independent modulation of the binding energy for specific intermediate, in terms of catalyst development and eletrolyte engineering.

## Strategies to tune intermediate binding

### Oxygen incorporation

Electrochemically reduced copper oxides, i.e. oxide-derived Cu (OD-Cu), have been intensively studied as an electrocatalyst for the production of C_2+_ chemicals from CO_2_RR [20, 24, 31], since Li et al. [32] first reported that the OD-Cu electrode can promote C–C coupling reactions with the suppression of HER in CO reduction reactions (CORR). It is known that the C–C coupling reactions in CO_2_RR actually proceed via similar pathways with those of CORR, such as CO dimerization [33, 34], therefore the OD-Cu electrode can show the enhanced C–C coupling capability even in CO_2_RR. The promotional effect of the OD-Cu electrode has been explained based on several hypotheses. Feng et al. [35] demonstrated that surface density of grain boundary is correlated with the catalytic activity of CO reduction, suggesting that defective grain boundary formed by oxidation treatment is an active site for C–C coupling reactions. Meanwhile, Xiao et al. [36] showed from DFT calculations that the Cu_2_O-Cu interface can lower the energetic barrier of C–C bond formation by attractive electrostatics between two carbon atoms bound to Cu^+^ and Cu^0^ atoms, respectively. Furthermore, it has been proposed that subsurface oxygen (O_sub_) plays an important role in modulating the binding strength of CO molecules which are known to be a key intermediate for C–C coupling reaction [20]. Here, we explain the promotional effect of OD-Cu electrodes focusing on the subsurface oxygen.

**Fig. 2:**
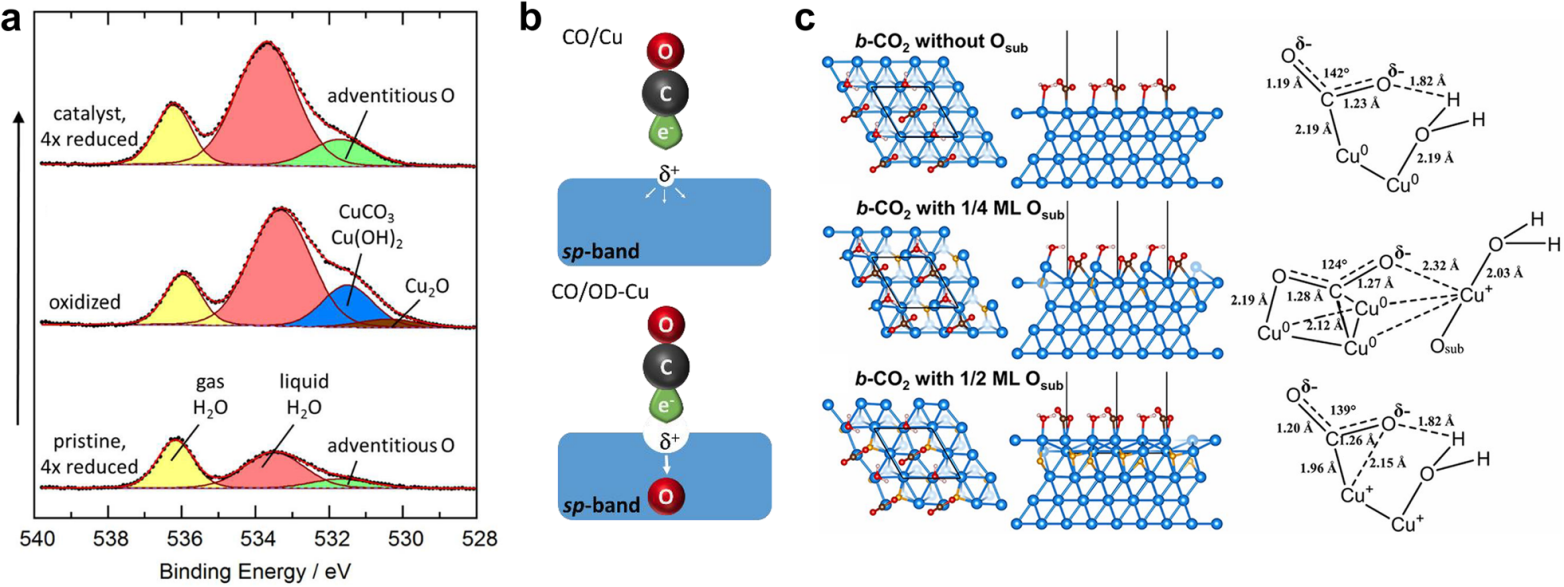
The direct observation of subsurface oxygens (O_sub_) and their effects on intermediate binding. **a** In situ O 1 s APXPS spectra of Cu foil depending on electrochemical reduction/oxidation treatments. Oxidation of the reduced Cu foil leads to the formation of a thick water overlayer and oxidized compounds such as CuCO_3_, Cu(OH)_2_, and Cu_2_O. Initially oxidized and then reduced Cu foil contains more adventitious oxygen (green). **b** Illustration of CO binding on Cu and oxide-derived Cu (OD-Cu) with O_sub_. **c** Predicted structures of bent CO_2_ (*b*-CO_2_) and H_2_O molecules on Cu(111) with different levels of O_sub_. Free energies for CO_2_ activation to *b*-CO_2_ are calculated to be +1.07, − 0.06, +0.28 eV on pristine Cu(111), Cu(111) with 1/4 ML O_sub_, and Cu(111) with 1/2 ML O_sub_ **a**–**b** (Reproduced with permission from [20], copyright 2017 American Chemical Society. **c** Reproduced with permission from [37], copyright 2017 National Academy of Sciences)

Eilert et al. [20] performed in situ ambient pressure X-ray photoelectron spectroscopy (APXPS) analysis to identify that oxygen species can be stably maintained at the surface even after applying cathodic potentials enough to reduce Cu oxides. Figure 2a shows the O 1 s APXPS spectra of the initial, oxidized, and reduced Cu foil. After oxidation, much thicker water overlayer was formed and oxidized compounds assignable to CuCO_3_, Cu(OH)_2_ and Cu_2_O were created. After re-reduction, they found that an oxygen peak increased much more in comparison with the initial spectrum of Cu foil. They hypothesized that the enhanced oxygen peak can be attributed to the formation of O_sub_, based on the peak position and additional oxygen K-edge electron energy-loss spectra (EELS) analysis.

In order to elucidate the influence of O_sub_ in CO_2_RR properties, the CO binding energy was calculated on clean Cu (100) and on Cu (100) with subsurface oxygens in different layers below surface. The CO binding became stronger in the presence of subsurface oxygens. Such enhancement of CO binding was explained by local charge interaction between O_sub_ and a Cu atom as shown in Fig. 2b. The O_sub_ could withdraw electrons from Cu sp-band, and lower the σ-repulsion between a Cu atom and CO molecule. Ultimately, it was suggested that stronger CO binding leads to a higher CO coverage at the catalyst surface, and kinetically promotes C–C coupling reactions over hydrogenation reactions such as CH_4_ and H_2_ evolutions.

It is also reported that the O_sub_ can lower the activation barrier for the transition from a physisorbed linear CO_2_ (*l*-CO_2_) to a bent chemisorbed CO_2_ (*b*-CO_2_). Favaro et al. [37] predicted the binding structures of chemisorbed CO_2_ and H_2_O on Cu (111) with different levels of O_sub_ (Fig. 2c). Also, they identified that the free energies of the chemisorbed systems depend on O_sub_ levels. When 0.25 mono-layer (ML) O_sub_ was incorporated, the free energy was much lowered in comparison with unmodified Cu (111). The presence of O_sub_ generated a heterovalent surface composed of Cu^0^ and Cu^+^ atoms, where the Cu^0^ and Cu^+^ centers were synergistically bound to the C and O atoms of the *b*-CO_2_, respectively. The electronic interactions between the binding centers and the adsorbed species stabilized *b*-CO_2_ structure significantly.

However, there are still on-going discussions as to whether the lattice oxygen is stable under highly cathodic conditions. Mandal et al. synthesized Cu_2_O catalysts and investigated their reduction behavior using in situ Raman spectroscopy [38]. They found that Cu_2_O signals completely disappeared during initial 3 min at CO_2_RR conditions, and CO_2_ reduction products were detected after complete reduction by selected-ion flow tube mass spectrometry (SIFT-MS). The experimental result was also supported by DFT calculation that Cu_2_O reduction is energetically preferred over CO_2_RR. Moreover, Lum et al. [39] also identified from ^18^O labelling experiments where the oxygen species of OD-Cu catalysts were observed to survive less than 1% of the original oxygen content. In contrast, it has been proposed that the O_sub_ can be stably sustained in (100) surfaces of Cu nanocubes and amorphous surface layers due to their disordered nature [40, 41], although O_sub_ is not stable in ordered Cu slab models. In fact, it has been reported that loss of Cu cube morphology by roughening during CO_2_RR caused the absence of Cu^+^ signals and the increase in HER over CO_2_RR [42]. The aggregated amorphous nanoclusters of Cu_2_(OH)_3_Cl were also found to have the mixed metal valency of Cu^+^ and Cu^0^ at highly cathodic potentials [33].

### Electrolyte engineering

**Fig. 3:**
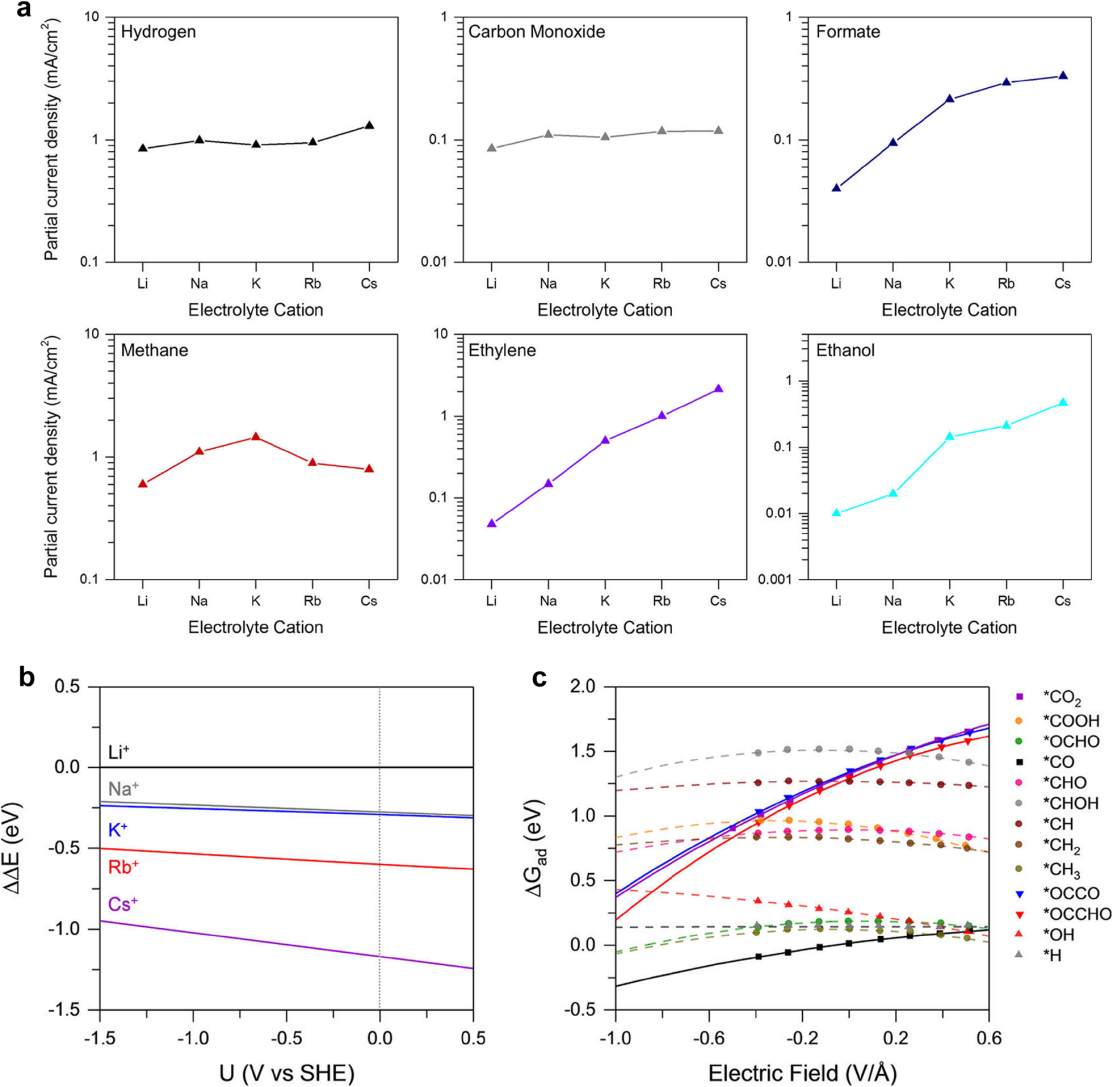
Cation effects on the stabilization of reaction intermediates. **a** Partial current densities for H_2_, CO, HCOO^−^, CH_4_, C_2_H_4_ and C_2_H_5_OH production on Cu (111) as a function of alkali metal cations included in an electrolyte. **b** The energy change occuring from the transport of a solvated cation from bulk electrolyte to the outer Helmholtz plane at the Cu (111) surface as a function of potential and cation type. **c** The effect of electric field on the adsorption free energies of various CO_2_RR intermediates on Cu (111) (**a**–**c** Reproduced with permission from [23], copyright 2017 American Chemical Society)

The cations and anions contained in an aqueous electrolyte also play an important role in controlling the product selectivity. Resasco et al. [23] measured the partial current densities of CO_2_RR on Cu(111) using different alkali metal cations such as Li^+^, Na^+^, K^+^, Rb^+^ and Cs^+^ as shown in Fig. 3a. Interestingly, as the cation size became larger, the partial current densities for HCOO^−^, C_2_H_4_ and C_2_H_5_OH production increased while the production rates of H_2_, CO and CH_4_ were unchanged. Larger cation size facilitated the CO_2_ activation over HER as well as the C_2_ production over C_1_. The effect of cation on product distribution was explained by electrostatic interactions between the intermediates and the solvated cations at the outer Helmholtz plane. Based on DFT calculation, it was demonstrated that larger cations are more stabilized in the outer Helmholtz plane at the Cu (111) surface (Fig. 3b), and show higher coverage near the catalyst surface. The stabilized cations facilitated the adsorption of intermediates with large dipole moments (e.g., *CO_2_, *CO, *OCCO) over *H intermediate with a negligible dipole moment through an electric field (Fig. 3c).

It has been also suggested that the other role of hydrated cations is to control the local CO_2_ concentration near the catalyst surface. Singh et al. [43] reported that cation hydrolysis occurs near the cathode surface during CO_2_ reduction. As the cation migrates toward the negatively polarized electrode, an increasing electrostatic interaction can reduce the p*K*_a_ for hydrolysis. If the local pH is higher than the p*K*_a_ of the hydrated cations, the dissociation of a water in the hydration shell releases protons, and buffers the change of local pH. The buffering ability declines in the order of Cs^+^ > Rb^+^ > K^+^ > Na^+^ > Li^+^. As local CO_2_ concentration competes with local pH due to the equilibrium reactions between CO_2_, HCO_3_^−^ and CO_3_^2−^ species, the pH decreases and the CO_2_ concentration increases near the cathode with increasing cation size.

Furthermore, Dinh et al. [44] found that higher concentration of OH^−^ ions can facilitate CO_2_ activation on the Cu-coated gas diffusion layer (GDL) electrodes. When linear sweep voltammetry (LSV) curves under CO_2_ and Ar flow were scanned in the electrolytes of various KOH concentrations, the onset of LSV curves recorded under CO_2_ shifted markedly to more positive potentials with increasing KOH concentration, whereas the onset under Ar remained similar. Furthermore, at 10 M KOH concentration, the onset potentials for CO and C_2_H_4_ production were similar, whereas the onset potential gap between CO and C_2_H_4_ is observed at low KOH concentration because the build-up of CO coverage is required to proceed CO dimerization. Therefore, high KOH concentration made CO dimerization much easier. Based on DFT calculation, they proposed that the hydroxide adsorption increases the charge imbalance between carbon atoms in the *OCCO intermediate. As a result, the dipole interaction between the carbon atoms more stabilized the *OCCO, and reduced the activation barrier for CO dimerization step.

### Nanostructuring

**Fig. 4:**
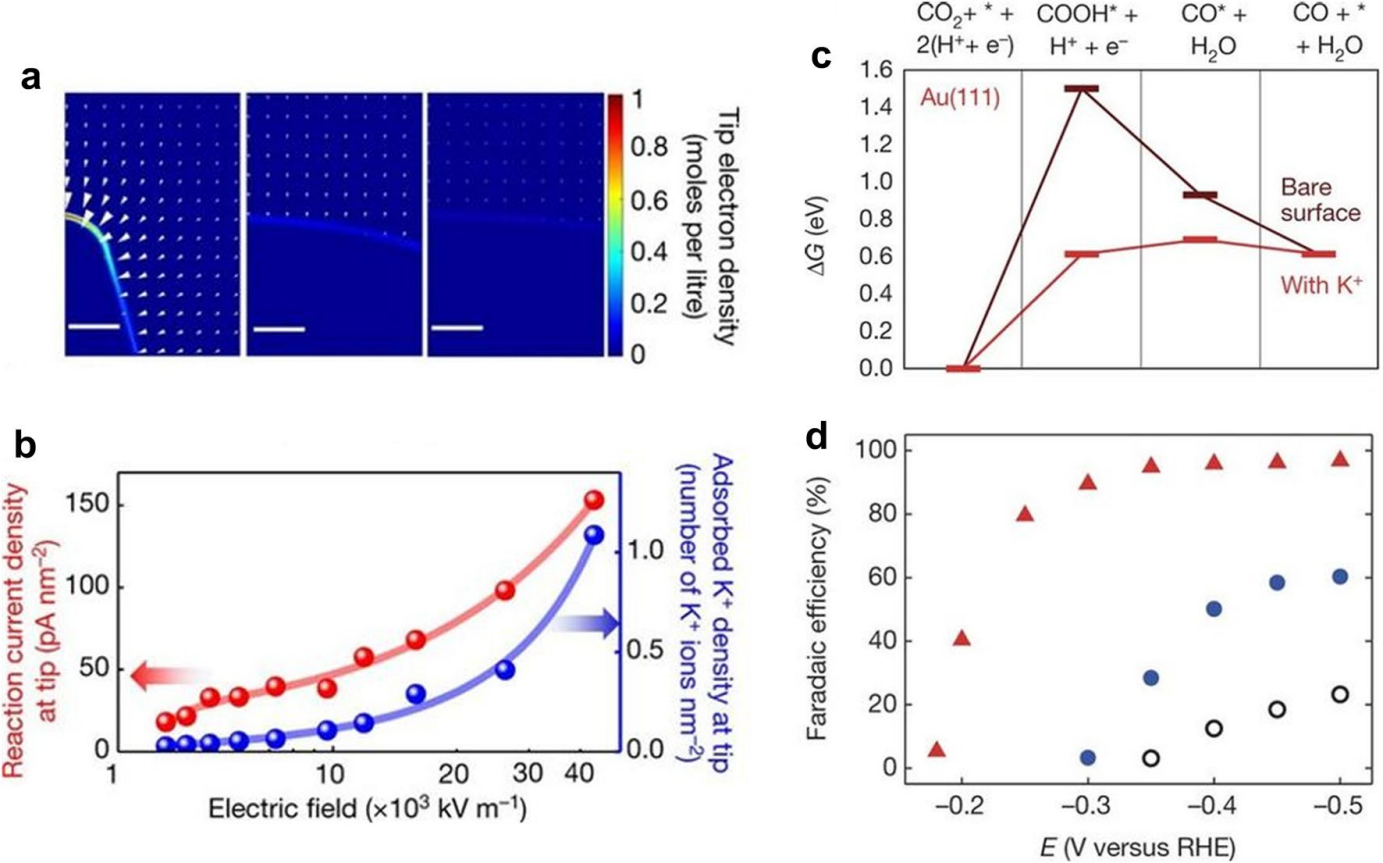
Electric field enhancement and the impacts on CO_2_ reduction properties on nanoneedle catalysts. **a** Free electron density distribution on the Au electrodes with different tip radius. The tip radius of the structure is 5, 60 and 140 nm for left, middle and right panels. **b** Adsorbed K^+^ density and current density distributions on the surface of Au needles. **c** Free energy diagrams of the electrochemical CO production on Au (111) surface in the presence of adsorbed K^+^ and in the absence of adsorbed K^+^. **d** CO FE on Au needles, rods and particles at different applied potentials (**a**–**d** Reproduced with permission from [22], copyright 2016 Nature publishing)

Sharpening metal can perturb the local electron density of metal surface and enhance the local electric field because free electrons migrate to sharp regions due to electrostatic repulsion. The field enhancement effect by tip electrode can be exploited for improving the CO_2_RR properties. Liu et al. [22] simulated the electron density distribution on various electrode surfaces as a function of tip radius (Fig. 4a). It was shown that the tip-concentrated electron density dramatically increases as the electrodes sharpen. The tip sharpening from a radius of 140 to 5 nm enhanced electrostatic field intensity from approximately 3 to 43 MV m^−1^. The enhancement of electric field also affected the concentration of surrounding alkali metal cations. The adsorbed K^+^ density at the tip elevated 20-fold due to the field enhancement (Fig. 4b). The highly concentrated cations can help to tune the free energy of adsorption of intermediates of CO_2_RR through the electrostatic interaction. According to DFT calculation study, the K^+^ ions greatly lowered free energy of *COOH and *CO adsorption, in comparison with the case without K^+^ ions (Fig. 4c). Based on these theoretical results, they attempted to measure the electrochemical CO_2_ reduction properties of Au needles, rods and particles. Surprisingly, Au needle electrodes exhibited a dramatic enhancement in CO_2_RR selectivity, as well as the onset potential shift to lower overpotentials (Fig. 4d). From Kelvin probe atomic force microscopy and inductively coupled plasma optical emission spectrometry (ICP-OES), it was also confirmed that electric fields and adsorbed K^+^ concentration are the highest for the needles.

Controlling the shape of catalysts has been reported to be effective even in C_2+_ production. Jiang et al. [45] prepared a Cu nanocube catalyst with exposed (100) facets through Cu^2+^ ion cycling method on Cu foil. When the cycle number increased from 0 to 100 cycles, the FE of C_2+_ products was dramatically enhanced from 26.0 to 60.6%. Concurrently, the FE of C_1_ products decreased from 59.1 to 22.7%. The enhanced C–C coupling capability was attributed to the exposure of (100) facets in addition to local pH effect resulting from high surface area because activity enhancement in the C_2+_ production was observed in low overpotential regions where mass transport effects can be neglected. According to DFT calculation, the Cu (100) surface showed stronger CO binding as well as lower CO dimerization barrier compared to Cu (111) surface. As a result, higher selectivity toward C_2+_ products was achieved. In addition to the facet-controlling strategy, constructing nanocavities at the catalyst surface can promote C_2_–C_1_ coupling to form C_3_ products. Zhuang et al. [34] simulated the spatial concentrations and flux distribution of CO, C_2_ and C_3_ species using a hollow spherical shell with a circular opening. They found that the inner cavity confines the mass transport of produced C_2_ species into exterior electrolyte, which results in higher local concentration of C_2_ species inside the cavity. Ultimately, the cavity structure increased surface coverage of C_2_ intermediate, and elevated the production rate of C_3_ species. To experimentally demonstrate the cavity effect, Cu catalysts with the cavity structure were prepared through acidic etching of Cu_2_O nanoparticles and then electrochemical reduction. The nanocavity Cu catalyst exhibited the enhanced FE of 21.0% towards propanol production compared to closed Cu nanoparticles (NPs), which was in accordance with the simulation results.

### Surface ligand effects

**Fig. 5:**
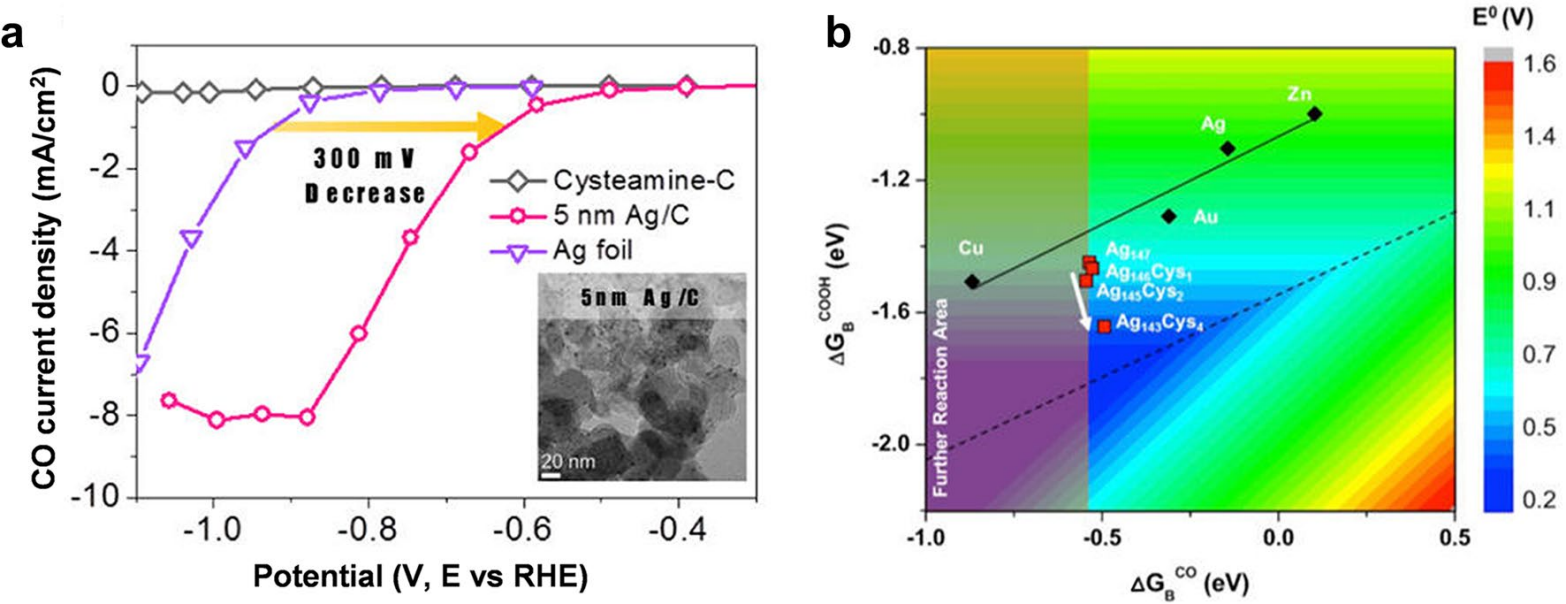
The effects of introducing ligand on the metal surfaces. **a** CO partial current density of cysteamine-anchored Ag/C, Ag foil, and cysteamine-C samples. **b** The binding energies of the COOH and CO intermediates examined from Ag_(147-n)_Cys_n_ (n = 0, 1, 2, 4) models. Colored map represents the theoretical CO_2_ reduction potential (E^0^) as a function of COOH and CO binding free energies (ΔG_B_^COOH^ and ΔG_B_^CO^) (**a**–**b** Reproduced with permission from [46], copyright 2015 American Chemical Society)

The ligand assembly can induce charge rearrangement, causing the difference in the intermediate stabilization and the adsorption configuration [46–48]. Kim et al. [46] synthesized Ag NP supported by carbon black in the presence of cysteamine as a ligand, and evaluated the electrocatalytic performance. The cysteamine-anchored Ag NPs showed a higher maximum FE of 84.4% toward CO production in comparison with 70.5% FE of Ag foil. More importantly, the overpotential required to achieve the CO partial current density of 1 mA cm^−2^ was reduced as 300 mV by the ligand assembly (Fig. 5a). By DFT calculation study, it was found that the binding energy of *COOH intermediate monotonically increases as the cysteamine coverage increases, while the CO binding energy exhibited marginal changes from the ligand assembly (Fig. 5b). Specifically, the binding energy of *COOH was enhanced up to 0.26 eV as the cysteamine/Ag ratio increases from 0 to 2.7%. The preferential stabilization of *COOH over *CO led to the reduction of overpotential. Deeper mechanistic insight was taken from the spatial electron spin density analysis. When cysteamine molecules were attached, spatial localization of the unpaired electron was stabilized particularly at the Ag surface, whereas the unpaired electrons were delocalized over the entire Ag atoms without the cysteamine. The unpaired electrons made the Ag–COOH bond more covalent, and stabilized the *COOH as a radical form.

The ligand effect has been also demonstrated with the different type of ligand for Au NP. Cao et al. [48] successfully prepared the porphyrin ligand-capped and naked Au NPs (denoted as P1-AuNP and naked-AuNP, respectively). The P1-AuNP showed higher electrocatalytic activity toward CO production compared to the naked-AuNP. At − 0.45 V vs. RHE, the CO FE was measured to be 93% and 44% for P1-AuNP and naked-AuNP, respectively. Moreover, at − 0.45 V vs. RHE, the partial current density for CO production was approximately sixfold higher on P1-AuNP than on naked-AuNP. Regarding the effect of porphyrin ligand, DFT calculation study showed that the formation of *COOH and *CO intermediates is more favorable as 0.55 and 0.08 eV on P1-Au(111) surfaces than on Au(111) surfaces. The porphyrin ligand dramatically lowered the energetic barrier for *COOH formation step.

### Single-atom catalyst

**Fig. 6:**
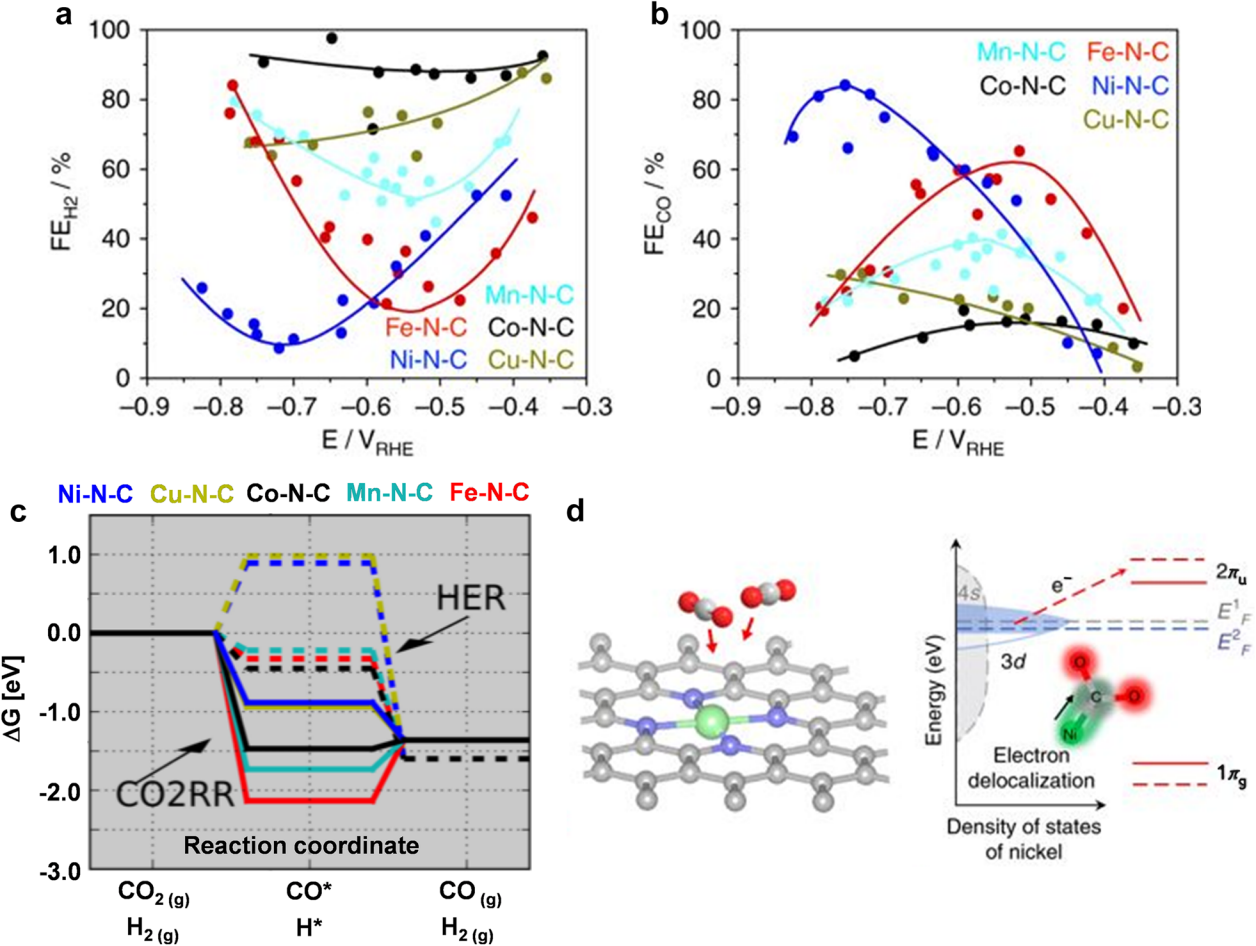
The electrochemical CO_2_ reduction properties of metal-incorporated N-doped carbon (M–N–C) catalysts. **a**, **b** The FEs of M–N–C (M = Mn, Co, Fe, Ni and Cu) at various potentials. **c** Free energy diagrams for the HER (dashed paths) and CO_2_RR (solid paths) at − 0.8 V vs. RHE for each M–N–C catalyst. **d** The schematics of CO_2_ activation process on the Ni(I) site. The red arrow indicates that electron transfer occurs from the Ni(I) to adsorbed CO_2_ (**a**–**c** Reproduced with permission from [51], copyright 2017 Nature Publishing Group. **d** Reproduced with permission from [52], copyright 2018 Nature Publishing Group)

Recently, single-atom catalysts have attracted great attention in the research field of electrochemical CO_2_ reduction due to their totally different activity compared to conventional bulk metals [49, 50]. One of the representative single-atom catalysts is metal-incorporated N-doped carbon (M–N–C) where a metal atom is coordinated by neighboring nitrogen atoms. Ju et al. [51] investigated the catalytic activity of M–N–C including various metals such as Mn, Co, Fe, Ni and Cu. They identified that the selectivity for CO production exhibit a strong dependence on the nature of metal center (Fig. 6a, b). The Co–N_x_ sites selectively produced H_2_ with the FE of 90% while the Ni–N_x_ sites showed the highest selectivity of 85% for CO production. To understand the experimental trend, DFT calculation study was conducted (Fig. 6c). It was discovered that Co–N_x_ sites have energetic downhill for H_2_ production, but represent too strong *CO adsorption to desorb the *CO. In contrast, on the Ni–N_x_ sites, the *H adsorption was energetically uphill reaction. Moreover, the formation and desorption process of the *CO intermediate had all-downhill energetics. In terms of the CO production rate, the Ni–NC catalyst outperformed the state-of-the-art Au catalysts. The CO_2_RR property of the Ni–NC catalyst is interesting, given that bulk Ni foil is specific for H_2_ production.

The special catalytic performance of single-atom metal catalysts can be attributed to the unique electronic structure of the metal center affected by surrounding coordination atoms. According to a previous study [52], it was explained that the unstable Ni(I) atoms with an unpaired electron can be stabilized by pyridinic N with low electronegativity in an N-doped carbon matrix. The Ni(I) site can transfer charges spontaneously to the carbon 2p orbital in CO_2_, forming a CO_2_^δ−^ species (Fig. 6d). Such an electronic interaction helps to reduce the energy barrier for electrochemical CO_2_ reduction. However, it should be also considered that various N species present in the N-doped carbons can act as an active site, in addition to M–N_x_ sites. Jung et al. investigated the catalytic performance of various M–N–C (M = Fe, Co, Cu) catalysts for CO_2_RR [53]. The maximum FE for CO production increased in the order of Cu–NC < Co–NC < Fe–NC. The maximum FE values were considered to be related with the atomic contents of pyridinic, pyrrolic, and graphitic N species based on XPS analysis. The catalysts containing higher pyridinic and graphitic N contents with lower pyrrolic N contents showed a higher selectivity toward CO production. Previous DFT calculation study also proposed that the N species in N-doped carbon nanotubes (NCNTs) can lower the energy barrier for *COOH formation because the N defects have lone-pair electrons and can donate electrons to the intermediate with strong binding, although the promotional effect depends on the type of N species [54].

## Conclusions

In summary, we have reviewed recent strategies for enhancing electrocatalytic properties of CO_2_ reduction reactions, and discussed new insights in terms of intermediate binding energy based on the DFT calculation results. Theoretically, suppressing the H_2_ evolution and improving the selectivity for a specific product on pure metal surfaces are limited by scaling relations between the binding energies of reaction intermediates, whereas it has been demonstrated that the binding energies can be tuned independently by implementing electronic perturbation and electrostatic interaction. Specifically, incoporating oxygens into the Cu surface stabilized the bent chemisorbed CO_2_ and strenghthened the CO binding, which kinetically promoted C–C coupling reaction for the production of C_2_H_4_ and C_2_H_5_OH. The utilization of larger alkali metal cations included in the electrolyte selectively enhanced the adsorption of intermediates with large dipole moments such as *CO_2_, *CO and *OCCO. In addition, it was revealed that the field enhancement by tip electrode increased the concentration of surrounding alkali metal cations, dramaticlaly lowering the adsorption free energies of *COOH and *CO. Furthermore, introducing surface ligand on Au and Ag NPs stabilized *COOH over *CO, which greatly elevated the selectivity and reduced the overpotential toward CO production. Also, the single-atom catalysts like Ni–N–C showed the high CO selectivity which cannot be achieved from the bulk Ni foil owing to strong *CO binidng. We believe that this review not only provide deep understandings of CO_2_ reduction reactions, but also help to create new ideas for future electrocatalyst developments.

## Abbreviations

HER: hydrogen evolution reactions
CO_2_RR: CO_2_ reduction reactions
DFT: density functional theory
OD-Cu: oxide-derived Cu
O_sub_: subsurface oxygen
APXPS: ambient pressure X-ray photoelectron spectroscopy
EELS: electron energy-loss spectra
*l*-CO_2_: a physisorbed linear CO_2_
*b*-CO_2_: a bent chemisorbed CO_2_
SIFT-MS: selected-ion flow tube mass spectrometry
GDL: gas diffusion layer
LSV: linear sweep voltammetry
ICP-OES: inductively coupled plasma optical emission spectrometry
M–N–C: metal-incorporated N-doped carbon
P1-AuNP: porphyrin ligand-capped Au NPs
NCNTs: N-doped carbon nanotubes
